# Enzymatic Management of Facial Overfilled Syndrome: A Case Series and Narrative Review

**DOI:** 10.1111/jocd.70377

**Published:** 2025-08-09

**Authors:** Desiree Castelanich, Luis Alberto Parra, Andreina Martinez Amado, Andrea Acevedo, Lina Velasquez, Valentina Dicker, Andrea Marcela Parra

**Affiliations:** ^1^ Sociedad Argentina de Dermatología Buenos Aires Argentina; ^2^ Sociedad Internacional de Rejuvenecimiento Facial no Quirúrgico (SIRF) Barranquilla Colombia; ^3^ Clinical Research – Encefalo Bogota Colombia; ^4^ Universidad del Rosario Cali Colombia; ^5^ Universidad del Valle Cali Colombia; ^6^ Universidad del Rosario Bogota Colombia

**Keywords:** collagenase, hyaluronidase, lipase, overfill face, overfill syndrome, recombinant enzymes

## Abstract

**Background:**

Facial overfilled syndrome (FOS) is an increasingly recognized complication of injectable aesthetic procedures, characterized by undesirable facial volume and contour irregularities. While hyaluronidase is well‐established for managing hyaluronic acid (HA) filler complications, treating FOS caused by non‐HA fillers, such as fat and silicone, remains challenging.

**Objective:**

This study aims to present the results of a novel enzymatic therapy combining hyaluronidase, collagenase, and lipase for treating FOS in five patients with complications from HA, fat, and silicone fillers. Additionally, we conducted a narrative literature review of studies published between 2015 and 2024, focusing on FOS and its management.

**Methods:**

We performed a narrative literature review using PubMed and Google Scholar databases, identifying six key papers that specifically address FOS. Following this, we present a case series of five patients treated with a combination of 1.5 mL collagenase, 1.5 mL hyaluronidase (HASA), and 1.5 mL lipase (total volume: 4.5 mL). Enzymatic therapy was administered via cannula under ultrasound guidance, and outcomes were assessed through clinical evaluation and patient‐reported satisfaction.

**Results:**

All five patients significantly improved facial volume and contour restoration after a single treatment session. The enzymatic combination effectively addressed complications from HA, fat, and silicone fillers, with no significant adverse events reported. Patient satisfaction improved significantly, highlighting the psychological benefits of the treatment.

**Conclusion:**

Combining hyaluronidase, collagenase, and lipase represents a safe and effective approach for managing FOS caused by various filler types. Our study, supported by a narrative review of six key papers on FOS, underscores the importance of tailored enzymatic therapies in aesthetic medicine and calls for further research to optimize treatment protocols and expand the evidence base.

## 1 Introduction

Aesthetic medicine integrates medical treatments and pharmaceutical products to improve physical appearance. This discipline has expanded due to technological advances, societal shifts, longer life spans, and a cultural emphasis on youthfulness [1]. Patients increasingly choose noninvasive procedures like fillers and laser treatments to maintain a youthful appearance and counteract aging [2]. According to the International Society of Aesthetic Plastic Surgery, there was a 6.5% rise in injectable procedures between 2019 and 2023 [3]. The injectable filler market is expected to expand from 3.4 billion to approximately 5.2 billion by 2026 [4].

However, as the popularity of injectable procedures grows, so does the risk of complications and adverse effects. One such condition is facial overfilled syndrome (FOS), characterized by an unnatural appearance caused by the excessive use of soft tissue fillers, particularly in the midface region [5, 6]. Despite being widely described in the scientific literature, no standardized diagnostic tool or objective measurement is currently used to confirm FOS. Diagnosis relies heavily on the expert eye of the clinician and the correlation between facial volumes [7]. The absence of standardized diagnostic criteria for FOS poses a significant challenge for clinicians, as it relies on subjective assessment and clinical experience.

FOS often arises from attempts to address signs of facial aging through aggressive filler treatments, which can lead to rapid volume changes, skin tightening, and disruption of natural facial harmony [5, 6, 7].

Despite the growing prevalence of FOS, treating this condition remains challenging, particularly when different filler materials—such as HA, fat, or silicone—are involved [8]. Each filler type presents unique difficulties in dissolution and removal, complicating the restoration of natural facial aesthetics [5]. Current treatment options, such as manual massage, aspiration, or surgical intervention, often fall short in addressing the diverse manifestations of FOS [5, 8]. Another approach, the dynamic filling technique, involves injecting small filler while the patient repeatedly smiles during the procedure. This method, supported by anatomical studies, allows for real‐time assessment of facial dynamics, helping to avoid overfilling and ensuring a more natural appearance [5]. Additionally, holistic assessment and alternative approaches, such as energy‐based devices and polymer reinforcement of facial ligaments, have been proposed to minimize reliance on fillers and prevent FOS. These methods emphasize the importance of tailored treatment plans that consider individual anatomical features and the dynamic nature of facial expressions [5, 6, 7, 8].

To better understand the current landscape of FOS and its treatment, we conducted a narrative review of the literature with significant relevance to FOS, enzymatic treatments, and filler complications, trying to provide a comprehensive overview of the current knowledge on FOS and its management.

In addition to the literature review, we present our clinical experience treating five patients who developed FOS using different filler materials, including HA, fat, and silicone. These cases highlight the challenges of managing FOS in diverse clinical scenarios and demonstrate the effectiveness of tailored treatment approaches, including hyaluronidase, collagenase, and lipase, in restoring natural facial aesthetics. The positive outcomes observed in these patients underscore the importance of individualized treatment plans and the need for further research to optimize FOS management.

## 2 Results

A narrative review of the literature focusing on studies published between 2015 and 2024. Databases such as PubMed and Google Scholar were searched using keywords including “Facial Overfilled Syndrome,” “hyaluronidase,” “collagenase,” “filler complications,” and “aesthetic medicine.” Studies were selected based on their relevance to FOS, enzymatic treatments, and filler complications, while those focused on non‐facial areas or nonenzymatic therapies were excluded. This review is organized thematically to provide a comprehensive overview of the current knowledge on FOS and its management (Table 1).

**TABLE 1:** Papers included in the narrative review.

Paper title	Principal author	Year	Methods	Key points	Clinical implications
Exploring facial overfilled syndrome from the perspective of anatomy and the mismatched delivery of fillers	Ting‐Song Lim [6]	2024	Literature review, anatomical analysis, ultrasound imaging, MRI studies	Understanding facial anatomy and aging process crucial for safe filler use; tailored assessment and treatment plans are essential; minimizing filler use is key	Careful filler selection and injection techniques; consideration of ethnic and gender differences; alternative approaches to minimize filler use
Anatomy behind the facial overfilled syndrome: The transverse facial septum	Sebastian Cotofana [5]	2019	Clinical study (volunteers), anatomical dissection (cadavers), 3D surface scanning	Transverse facial septum explains FOS; dynamic filling technique recommended to avoid FOS	Avoid overfilling; consider the transverse facial septum during midface filler injections; use dynamic filling technique
Treating facial overfilled syndrome with impaired facial expression—Presenting clinical experience with ultrasound imaging	Leonie Schelke [8]	2023	Clinical study, ultrasound imaging, hyaluronidase injections	Ultrasound‐guided hyaluronidase is effective for treating facial overfilled syndrome, particularly the unnatural smile component	Ultrasound‐guided hyaluronidase injections are a safe and effective treatment option for facial overfilled syndrome. Careful assessment of filler location is crucial
How should we use hyaluronidase for dissolving hyaluronic acid fillers?	Gi‐Woong Hong [9]	2025	In vitro and in vivo experiments evaluating hyaluronidase's effectiveness on various HA fillers	Direct injection is superior; biphasic fillers dissolve faster; timely intervention is crucial due to short hyaluronidase half‐life; consider patient hypersensitivity	Tailor hyaluronidase dose and technique to filler type; use direct injection for optimal results; be prepared to manage potential allergic reactions; timely treatment is essential
Psychology of aesthetics: Beauty, social media, and body dysmorphic disorder	Melissa R. Laughter [10]	2023	Literature review	Understanding societal influences on beauty perception is crucial for managing BDD; careful assessment of patients for BDD is essential before cosmetic procedures	Screen patients for BDD; manage expectations regarding cosmetic procedures; utilize alternative image formats for consultations to minimize distortion
The overfilled face	Nabil Fakih [7]	2022	Literature review	Overfilled face syndrome is an aesthetic issue with potential psychological consequences; emphasis should be on natural results and avoidance of overfilling	Careful filler selection, precise injection techniques, and realistic patient counseling are critical; hyaluronidase can be used for correction; sometimes surgical intervention is necessary

## 3 Overview of FOS

The concept of FOS was introduced in the literature in 2016, when Pessa et al. identified the transverse facial septum and its role in the dynamics of facial expressions following soft tissue filler injections [11]. This foundational work laid the groundwork for understanding how excessive filler can lead to an unnatural appearance and impaired facial movement [5]. As described in the introduction, FOS is characterized by an unnatural appearance, often manifesting as “chipmunk” cheeks, “flowerhorn” foreheads, and “pillow” faces [5, 6]. These symptoms arise from aggressive filler treatments that disrupt the natural balance of facial anatomy, leading to rapid volume changes and skin tightening [8].

Historically, facial anatomy was understood in terms of distinct layers of fat and fascia. However, recent research, such as the work by Cotofana et al. in 2020, has emphasized the importance of vertical structures, particularly the transverse facial septum, in maintaining the natural dynamics of facial expressions [5].

Historically, facial anatomy was understood in terms of distinct layers of fat and fascia. However, recent research like the one presented for Cotofana et al. in 2020 indicates that vertical structures, such as the transverse facial septum (which plays a critical role in maintaining the natural dynamics of facial expressions) located beneath the zygomaticus major muscle, separate the buccal space from the deep midfacial fat compartments and is essential for normal facial movement [5]. Located beneath the zygomaticus major muscle, this septum separates the buccal space from the deep midfacial fat compartments and is essential for normal facial movement [5]. FOS can develop when fillers are injected without adequately considering the underlying facial anatomy and the dynamic nature of facial expressions [6, 8]. For example, in 2023, Lim et al. demonstrated that excessive filler in the midface can restrict the movement of facial muscles, leading to an unnatural appearance, especially when the patient smiles or makes other expressions [6].

The psychological impact of FOS is significant. The perception of beauty is fluid and can be heavily influenced by cultural practices, societal interactions, and exposure to social media [10]. Beauty ideals vary widely across cultures and are often shaped by environmental factors and perceptual adaptation [10]. Patients seeking cosmetic procedures may be influenced by idealized images on social media, leading them to pursue treatments that promise to enhance their appearance [10]. However, the pressure to conform to these ideals can result in overcorrection, where the desire for a youthful or attractive look leads to excessive filler use, ultimately resulting in FOS. This phenomenon underscores the importance of educating patients about the risks of overfilling and the need for individualized treatment plans that prioritize natural aesthetics.

## 4 Key Factors Contributing to the Occurrence of FOS

### 4.1 Excessive Filler Use

The primary cause of FOS is the over‐injection of fillers, particularly in the midface region [7]. Patients may seek to enhance their facial features significantly, leading practitioners to inject more filler than necessary. This can result in a heavy or distorted appearance, especially when the filler is not evenly distributed [7].

### 4.2 Inappropriate Injection Techniques

Improper injection techniques can exacerbate the problem. As described by Ting‐Song Lim et al. in 2023 [6] and Cofotana et al. in 2020 [5] injecting fillers without considering the underlying facial anatomy can lead to uneven distribution and excessive volume in certain areas [5, 6]. The lack of attention to the dynamic nature of facial expressions during the injection process can also contribute to FOS [7].

### 4.3 Anatomical Considerations

The facial anatomy plays a crucial role in the development of FOS [5, 7]. The transverse facial septum, which separates different fat compartments in the midface, is essential for normal facial movement and expression [5]. Excess filler can interfere with the mobility of this septum and the surrounding muscles, leading to an unnatural appearance when the patient smiles or makes other facial expressions, as explained by Cotofana et al. in 2020 [5].

### 4.4 Patient Expectations and Psychological Factors

Patients often have high expectations regarding the outcomes of filler treatments, as it was described in 2023 by Laughter et al. [10] Beauty perception usually is influenced by societal beauty standards and images seen on social media; this can lead to a desire for dramatic changes, prompting both patients and practitioners to pursue excessive filler use, which can ultimately result in FOS [10].

### 4.5 Cultural and Ethnic Factors

According to Lim et al., different ethnicities may have varying facial structures and beauty ideals, influencing how fillers are perceived and applied [6]. For instance, individuals of East Asian descent may be more susceptible to FOS due to specific anatomical characteristics, such as zygomatic arch protrusion, which can amplify the effects of overfilling [6]. These anatomical variations highlight the importance of tailoring filler treatments to individual facial features and ethnic backgrounds to achieve harmonious results. In addition, the influence of social media has significantly shaped beauty standards and patient expectations [10]. Filters that exaggerate cheek volume and chin projection have become increasingly popular, promoting an idealized yet often unrealistic aesthetic. This trend has led to a rise in overvolumized treatments, where the natural harmony of the three facial thirds is disrupted. The pressure to conform to these digitally enhanced ideals has contributed to the growing incidence of FOS, as patients seek dramatic transformations without fully considering the risks of overfilling [12].

## 5 Treatment Option for FOS

Patients presenting with FOS symptoms, such as an unnatural smile or excess midfacial volume, should start with a complete medical and anatomical evaluation using clinical examination and technological tools, such as facial ultrasound imaging. High‐frequency ultrasound or even more precise magnetic resonance or tomography may be a key factor to identify the anatomical location within facial layers, such as fat compartments or the transverse facial septum, ensuring targeted treatment planning of the filler [8]. Hyaluronidase, the primary treatment for dissolving hyaluronic acid (HA) fillers, is guided by factors, such as filler type, enzyme concentration, and application technique [13]. It is essential to highlight its role as a spreading factor, which allows hyaluronidase (HASA) to be used for complete HA dissolution and redistributing excess filler without entirely removing it [14]. Also, hyaluronidase can enhance fibrinolysis in cases involving other substances, helping reduce inflammatory reactions and fibrosis associated with foreign body injections [15]. This dual functionality makes hyaluronidase a versatile tool in managing complications related to both HA and non‐HA fillers [16]. According to Gi‐Woong Hong et al. in their publication 2025 [9], approximately 500 units of hyaluronidase per 1 mL of biphasic fillers is typically sufficient; in contrast, monophasic fillers, which are more cross‐linked, may require up to 750 units per 1 mL and multiple injections for effective dissolution. Direct injection into the filler mass is the most effective technique, allowing thorough penetration and faster breakdown [9]. Timely administration is critical, as hyaluronidase activity diminishes significantly within 30 min.

While using hyaluronidase remains the gold standard for treating FOS caused by HA fillers, managing complications from other filler materials, such as fat or silicone, presents unique challenges. To address these challenges, we report five female patients treated in Latin American countries between 2019 and 2024. These patients, aged 27–55, presented with FOS following treatments with various fillers, including HA, fat, and silicone. The overvolumization of their facial features significantly impacted their mood and self‐perception, prompting them to seek professional management. All patients provided informed consent and were authorized to use their photographs and clinical data for publication in international scientific journals.

## 6 Case Report

### 6.1 Patient Demographics and Clinical Presentation

**FIGURE 1:**
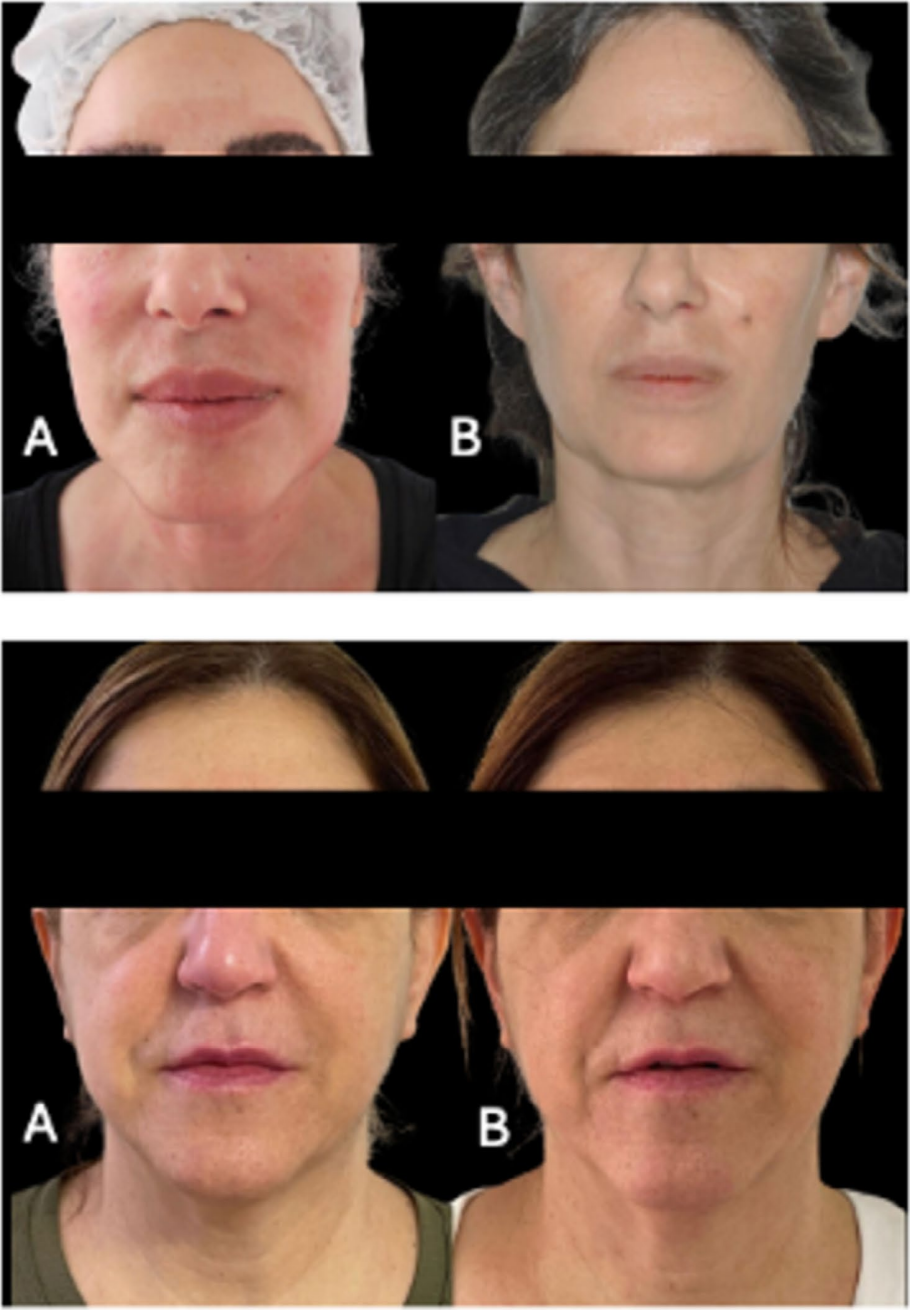
Patients 1 and 2, women who experienced facial overfilled syndrome (FOS) following hyaluronic acid (HA) filler treatments. (A) Pretreatment photographs showing overvolumization in the midface and lower face. (B) Posttreatment photographs after a single session of enzymatic therapy using a combination of 1.5 mL collagenase, 1.5 mL hyaluronidase (HASA), and 1.5 mL lipase (total volume: 4.5 mL), administered via cannula. The treatment resulted in significant improvement in facial volume, restoration of natural contours, and enhanced patient self‐confidence and comfort with their appearance.

**FIGURE 2:**
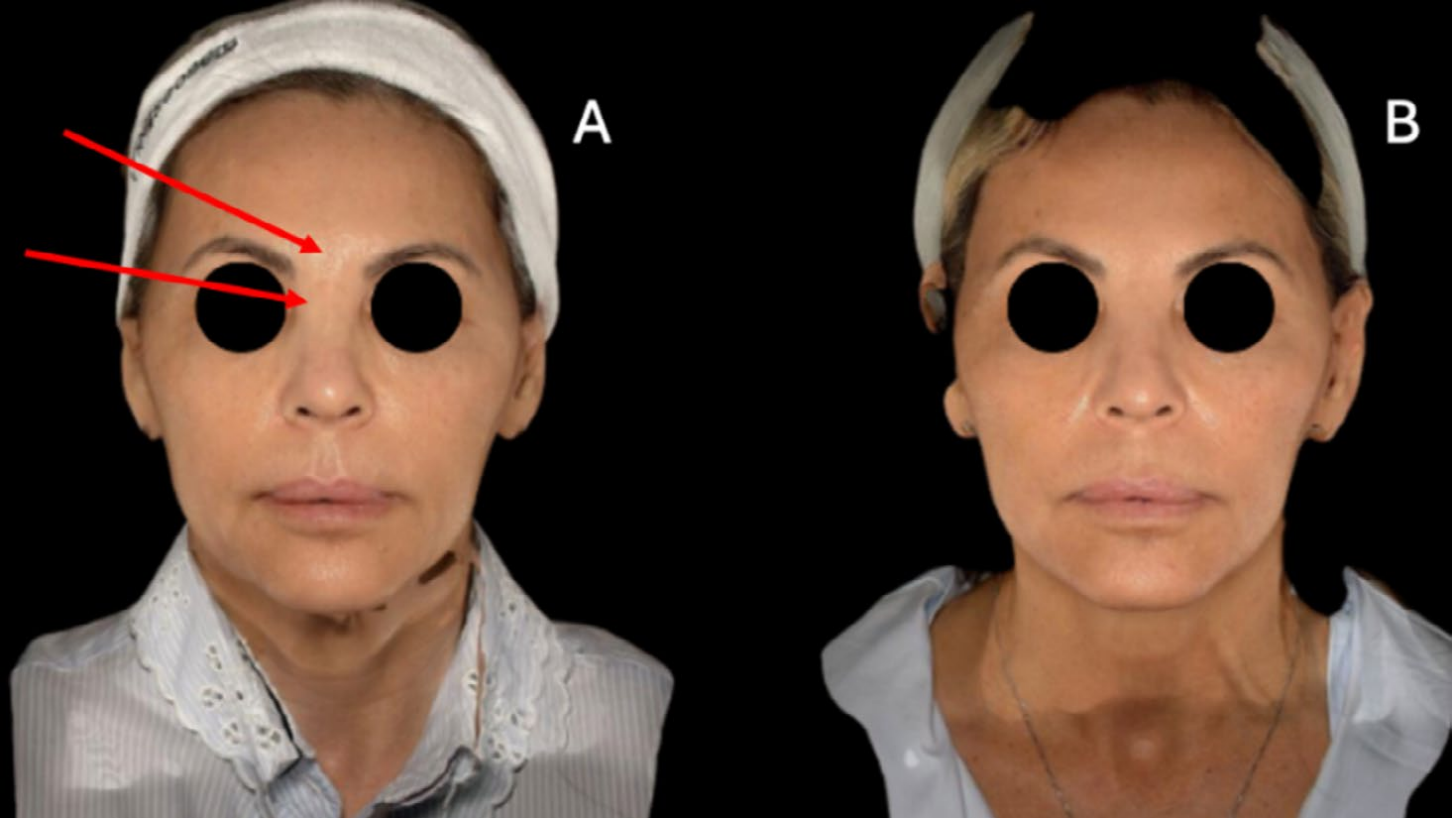
Patient 3, a 55‐year‐old woman who experienced facial overfilled syndrome (FOS) following hyaluronic acid (HA) filler injections in the cheeks and midface, as well as silicone filler in the glabella and nasal dorsum. (A) Pretreatment photographs showing overvolumization in the midface and nose. (B) Posttreatment photographs after a single session of enzymatic therapy using a combination of 1.5 mL collagenase, 1.5 mL hyaluronidase (HASA), and 1.5 mL lipase (total volume: 4.5 mL), administered via cannula. The treatment significantly improved facial volume and restored the natural look, especially on midline face, with better nose projection, and enhanced patient self‐confidence and satisfaction with their appearance.

**FIGURE 3:**
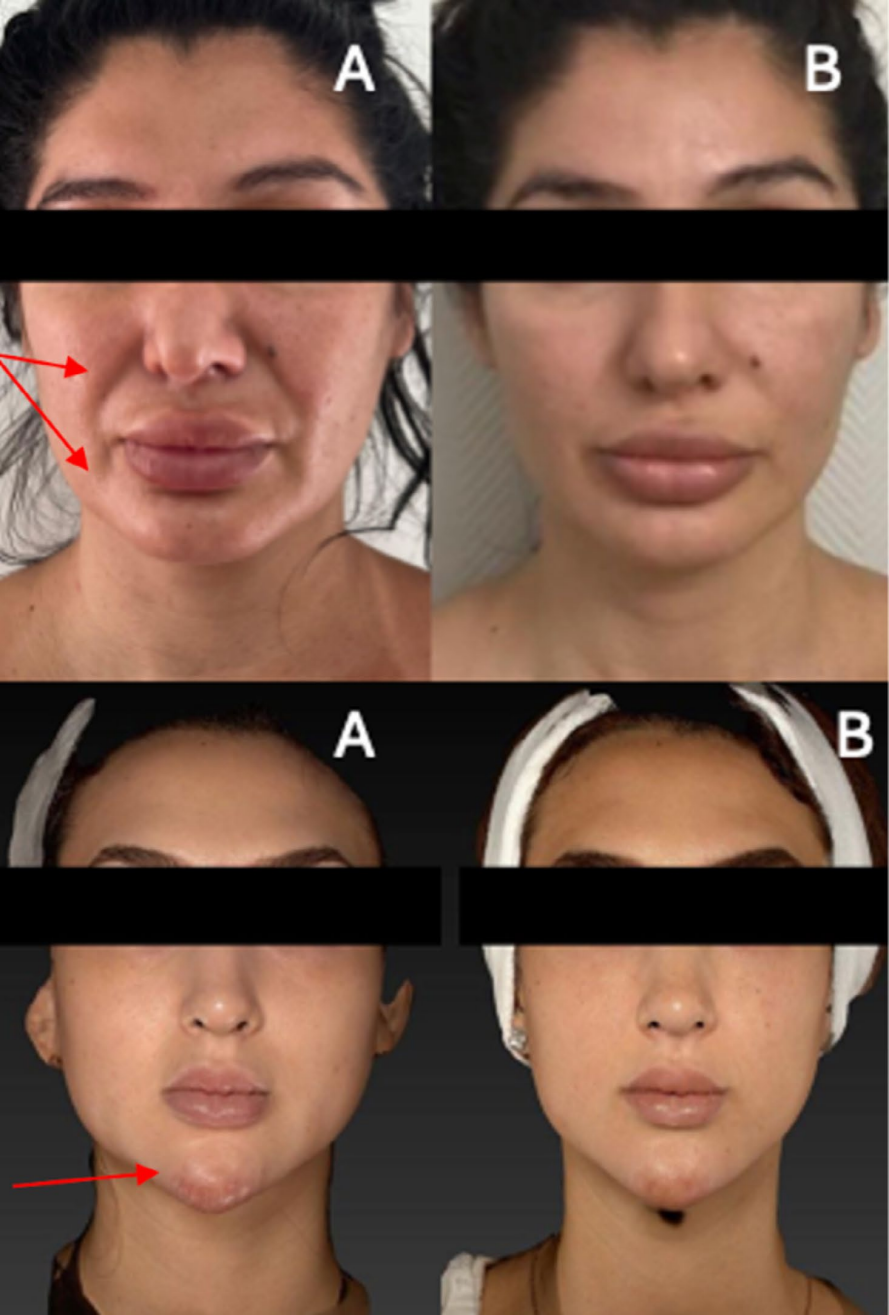
Two female patients: Patient 4 (35 years old) experienced facial overvolumization (FOS) following fat grafting. After fat grafting in the midface, the fat was positioned on the temporal area, nose, and nasolabial folds, and Patient 5 (27 years old) experienced FOS due to silicone‐based fillers in the chin and facial contours. Both patients received two sessions with 1‐month interval. Panel (A) shows the patients administered via cannula before treatment with a single combined enzyme therapy (1.5 mL collagenase, 1.5 mL hyaluronidase, and 1.5 mL lipase; total volume: 4.5 mL). Panel (B) shows the improvement in facial overvolumization after two treatment sessions, with 1‐month interval.

- Patient 1: A 38‐year‐old female with FOS following HA filler treatment in the midface (Figure 1).
- Patient 2: A 45‐year‐old female with FOS after HA filler injections in the cheeks and nasolabial folds (Figure 1).
- Patient 3: A 55‐year‐old female with FOS due to HA filler on her cheeks and middle face and silicon filler on the nasal dorsum and glabella, with overcorrection (Figure 2).
- Patient 4: A 35‐year‐old female with FOS, after fat grafting in the midface, the fat was positioned on the temporal area, nose, and nasolabial folds (Figure 3).
- Patient 5: A 27‐year‐old female with FOS caused by silicone‐based fillers in the chin and facial contour (Figure 3).

### 6.2 Treatment Protocol

#### 6.2.1 Enzymatic Composition

All patients were treated using a combination of 1.5 mL collagenase, 1.5 mL hyaluronidase (HASA), and 1.5 mL lipase (total volume: 4.5 mL), all recombinant enzymes from PBserum Medical, Madrid, Spain. The mixture was diluted with 1.5 mL of 0.9% sodium chloride.

#### 6.2.2 Administration Technique

The enzymatic mixture was administered via a cannula in the areas of the face with excessive volume, using fan technique in retroinjection following the patient's anatomy. Special care was taken to respect the transverse facial septum as a critical anatomical structure for optimal volume distribution, per Cotofana et al. [5]. The procedure was performed in a single session for Patients 1 and 2, and two sessions for Patients 3, 4, and 5 (due to silicone and fat filler complications).

### 6.3 Outcomes

Following treatment, all five patients showed marked improvement in facial overvolumization, including those with silicone‐based fillers (Figures 1, 2, 3). The reduction in excess volume restored natural facial contours and significantly improved patient satisfaction. No complications were reported, and all patients expressed relief from the psychological burden of FOS.

## 7 Discussion

FOS is an emerging complication associated with injectable aesthetic procedures [6, 8]. Characterized by undesirable facial volume and contour irregularities, it represents a significant challenge in cosmetic dermatology. Since its initial description in 2016 [11], our narrative search of the available literature reveals a scarcity of robust randomized controlled trials and meta‐analyses, limiting definitive conclusions regarding optimal treatment strategies. This paucity of high‐level evidence, particularly regarding the comparative efficacy of different treatment modalities, underscores the need for further research.

In this context, we present a case series of five patients treated with a novel enzymatic therapy combining hyaluronidase, collagenase, and lipase (PBserum Medical, Madrid, Spain). Our approach demonstrated excellent results in reducing overvolumization and restoring natural facial contours.

While hyaluronidase has been extensively studied and established as the gold standard for managing complications related to HA fillers, collagenase and lipase represent a significant advancement in the field. As previously demonstrated by the authors in a 2024 publication, combining collagenase and hyaluronidase can effectively improve periocular edema following HA filler injections [17]. These enzymes facilitate the degradation of HA into small, medium, or high molecular weight fragments by breaking the β‐1,4 glycosidic bonds [15]. Unlike bacterial hyaluronidases, which belong to the lyase group and do not act via hydrolysis, the resulting HA fragments exhibit variable biological activity. High molecular weight fragments demonstrate anti‐inflammatory, antiangiogenic, and potentially wound‐healing properties, while low molecular weight fragments are associated with angiogenic and inflammatory activity [15].

Collagenase, particularly the clostridium‐derived collagenase GH PB220, induces multiple cleavages along the collagen triple helix, leading to complete collagenolysis into small peptides [15]. Additionally, clostridial collagenases reduce the expression of fibrosis‐related molecules, such as fibronectin, smooth muscle actin, and transforming growth factor‐beta (TGF‐β), while sparing type IV collagen, which forms the basal lamina of blood vessels and nerves [18]. This selective action promotes the degradation of old and fragmented collagen while preserving the structural integrity of critical tissues [19], facilitating the formation of granulation tissue, decreasing inflammation, supporting epithelial cell regeneration, and accelerating wound healing. These properties underscore the potential applications of collagenase in enhancing skin appearance, treating scars, and preparing patients for surgical procedures [17, 18].

Engineered lipases facilitate the breakdown of triglycerides into glycerol and free fatty acids, as Gabriel A. Mecott‐Rivera et al. demonstrated in a porcine animal model. Lipase can be injected into the target area to promote the hydrolysis of excess fat [20]. This enzymatic action can reduce the size of adipocytes, thereby decreasing the overall volume in the treated area and mobilizing fat from adipocytes, leading to a decrease in adipocyte volume without causing significant inflammation or damage to surrounding tissues [20]. Furthermore, lipase can be integrated into combination therapies. For example, it may be used alongside collagenase and hyaluronidase in areas where both fat reduction and filler degradation are desired, as Jabbour et al. presented in 2024 [21].

Although hyaluronidase is extensively researched for HA filler complications [13, 16], defining optimal dosages and application mechanisms remains challenging. As Gi‐Woong Hong et al. demonstrated in their 2025 study – which evaluated hyaluronidase efficacy across HA filler types and proposed clinical guidelines via in vitro/in vivo experiments [9], standardization difficulties persist. To address this, we combined hyaluronidase with collagenase and lipase. This enzymatic approach resolved FOS in all five patients within one or two sessions, including a case with fat/silicone fillers requiring two treatments. We attribute these outcomes to reduced facial edema and improved overall aesthetics from the enzyme synergy.

This aligns with recent advancements in enzymatic treatments that suggest a combination of collagenase, hyaluronidase, and lipase, showing that it can improve, for example, fibrotic responses in cellulite treatment [22, 23, 24]. Collagenase breaks down collagen structures contributing to fibrosis, while hyaluronidase dissolves HA fillers. As Erick Santaella‐Sosa et al. described [22], a standardized three‐session protocol combining these enzymes can enhance buttock appearance without altering volume [22]. Similarly, Castelanich et al. demonstrated that combining hyaluronidase and collagenase effectively resolves delayed periocular edema following filler treatments while preventing post‐hyaluronidase syndrome [25].

Our findings have significant clinical implications, particularly for patients experiencing FOS due to non‐HA fillers, such as silicone, a non‐biological substance used in aesthetic procedures to enhance facial features and address signs of aging. Assis Machado et al.'s systematic review of 303 patients across multiple countries reported silicone filler usage in 19.7% of cases [26]. The most common histopathological finding associated with silicone fillers was foreign body granuloma, which is traditionally more challenging to manage [26].

While this narrative review and report of a case of a patient treated in Latin America demonstrates promising results, it is not without limitations. The small sample size of five patients limits the generalizability of our findings. Future studies with larger, more diverse cohorts must validate our conclusions and optimize the dosages and application techniques of collagenase, hyaluronidase, and lipase to enhance treatment outcomes.

## 8 Conclusion

FOS is a growing concern in aesthetic medicine, exacerbated by the influence of social media and the increasing demand for injectable treatments. When FOS occurs, it not only severely compromises patients' self‐esteem and confidence but also tarnishes the reputation of injectable procedures. This underscores the urgent need for innovative and effective treatment options to address this complication and provide injectors with reliable alternatives.

In this study, we first conducted a narrative review of the literature, involving all authors in the screening and selecting relevant articles. Our review revealed that FOS is rarely discussed as a distinct entity; instead, it is often grouped under broader categories of filler‐related complications. This lack of focused research limits the availability of evidence‐based management strategies, highlighting the importance of studies like ours to fill this gap.

We also present our clinical experience treating five patients with FOS using a novel combination of three recombinant enzymes: hyaluronidase, collagenase, and lipase. All patients showed significant improvement in facial appearance, with reduced overvolumization and restoration of natural contours. Importantly, our approach did not eliminate the silicone itself but improved associated edema, fibrosis, and water retention in the patient treated with silicone‐based fillers. This distinction is critical to avoid misinterpretation of our findings.

Finally, we emphasize the importance of case reports, controlled studies, and literature reviews to expand the available evidence on FOS. By sharing our experience and contributing to the growing body of literature, we hope to inspire further research and provide clinicians with the tools to manage this challenging complication effectively. Only through continued investigation and collaboration can we improve patient outcomes and uphold the safety and reputation of injectable aesthetic procedures.

## Author Contributions

**Desiree Castelanich:** conceptualization, data collection, manuscript drafting, and review. **Luis Alberto Parra:** patient treatment, data analysis, and manuscript review. **Andreina Martinez Amado:** statistical analysis, data interpretation, and manuscript editing. **Andrea Acevedo:** patient recruitment, treatment, and data collection. **Lina Velasquez:** data cleaning, validation, and manuscript editing. **Valentina Dicker:** statistical analysis, data interpretation, and manuscript review. **Andrea Marcela Parra:** manuscript drafting, editing, and review. All authors contributed to the final manuscript, reviewed, and approved the submitted version.

## Ethics Statement

This study was conducted in accordance with the ethical principles outlined in the Declaration of Helsinki. All patients provided informed consent prior to participation, including consent for the use of their clinical data and photographs for publication in scientific journals. The treatment protocols and procedures were performed with the utmost care to ensure patient safety and well‐being. No identifying information was disclosed, and patient confidentiality was maintained throughout the study. The research did not involve any experimental or unapproved treatments, and all procedures were performed by qualified medical professionals in compliance with ethical standards.

## Conflicts of Interest

The authors declare no conflicts of interest.

## Data Availability

The data that support the findings of this study are available on request from the corresponding author. The data are not publicly available due to privacy or ethical restrictions.
